# C-Reactive Protein Mediates the Association Between Subjective Aging and Incident Heart Disease

**DOI:** 10.1093/geroni/igab046.2310

**Published:** 2021-12-17

**Authors:** Hanamori Skoblow, Christine Proulx

**Affiliations:** University of Missouri, Columbia, Missouri, United States

## Abstract

Recent studies have shown that negative perceptions of subjective aging are associated with a heightened risk of cardiovascular events (Stephan et al., 2020) and increased C-reactive protein (CRP), a biomarker associated with inflammation (Stephan et al., 2014). Because inflammation is deleterious to cardiovascular health, CRP might mediate the association between subjective aging and cardiovascular disease. The purpose of this study was to examine the association between subjective aging (i.e., negative self-perceptions of aging [SPA] and subjective age) and incident cardiovascular disease (e.g., heart attack, angina, congestive heart failure), and to determine whether CRP mediates this relation. We used up to five waves of repeated measures data from the Health and Retirement Study (HRS, 2008 - 2016) with adults aged 50 to 101 (n = 9,531). Two separate models were conducted in MPlus with bias-corrected bootstrap confidence intervals and controls for respondent age, sex, education, race, ethnicity, body mass index (BMI), diabetes, hypertension, depressive symptoms, and physical inactivity. There were significant indirect effects of both SPA and subjective age on incident cardiovascular disease through CRP (indirect effect SPA model = .02, CIs [.01, .03], p < .05; indirect effect subjective age model = .05, CIs [.02, .10], p < .05). In both models, CRP fully mediated the association between subjective aging and incident cardiovascular disease. Taken together, these findings underscore the importance of considering older adults’ views of aging for understanding physical health and suggest that interventions aimed at improving views on aging may reduce inflammation and promote cardiovascular health.

